# Study on water use sources and ecohydrological niche characteristics of two dominant shrubs in the Tengger Desert

**DOI:** 10.3389/fpls.2026.1755414

**Published:** 2026-04-14

**Authors:** Longfei Zhao, Fucang Qin, Xiaoyu Dong

**Affiliations:** 1 College of Desert Control Science And Engineering, Inner Mongolia Agricultural University, Hohhot, Inner Mongolia, China; 2 Inner Mongolia Academy of Forestry Sciences, Hohhot, Inner Mongolia, China

**Keywords:** hydrogen and oxygen isotopes, MixSIAR, Tengger Desert, ecohydrological niche, water utilization

## Abstract

In arid regions with extremely limited water resources, understanding water-use strategies of dominant shrubs is critical for ecohydrology and vegetation restoration. This study investigated the water sources of *Nitraria tangutorum* and *Zygophyllum xanthoxylum* in the eastern Tengger Desert, using soil water monitoring and hydrogen-oxygen stable isotopes(*δ*²H, *δ*¹^8^O). Levins’ index and the Proportional Similarity (PS) index were used to quantify niche breadth and similarity in water-source use. Soil water content in *N. tangutorum* sites was generally higher than that in *Z. xanthoxylum* sites, particularly in the deep layers. The slopes of the local meteoric water line and soil water line were lower than the global meteoric water line, indicating evaporative enrichment in precipitation and soil water. *N. tangutorum* xylem water aligned more closely with shallow to middle layer soil water, whereas *Z. xanthoxylum* corresponded more closely to middle to deep soil water, demonstrating vertical differentiation in water uptake. MixSIAR results revealed that *N. tangutorum* primarily utilized water from the 40–200 cm soil layer in the early growing season, shifted to shallow soil water (0–40 cm) during the peak precipitation period, and subsequently shifted back to middle and deep water sources. In contrast, *Z. xanthoxylum* consistently relied on water from the 120–200 cm soil layer throughout the growing season, with a minor groundwater contribution. Both shrubs exhibited relatively broad ecohydrological niche breadths across the growing season. However, during the peak precipitation period in July and August, their niche breadths contracted, and the similarity index of soil water and groundwater utilization decreased. Overall, the two shrubs exhibited contrasting water-source use patterns: *N. tangutorum* showed greater seasonal flexibility, shifting toward shallow soil water during July–August, whereas *Z. xanthoxylum* maintained a relatively stable reliance on deeper soil water. Because sampling was conducted in species-specific stands rather than mixed-species plots, niche metrics are interpreted as comparative indicators of water-source use patterns under shared regional conditions. These findings provide a basis for species selection and water management for desertification control and vegetation restoration.

## 1 Introduction

Water is the key limiting factor governing vegetation distribution and growth in arid and semi-arid regions (Haddoudi et al., 2021). Plants absorb soil water through their root systems to meet the demands of survival and growth, and this process is jointly regulated by precipitation regimes, soil water-storage properties, and plant functional traits (Dai et al., 2022; Han et al., 2021). In environments with extremely scarce water resources—particularly deserts—plant survival largely depends on the pattern and efficiency of soil water use. Therefore, a detailed understanding of plant water-use strategies is essential for elucidating the adaptive mechanisms of dryland vegetation and for providing a scientific basis for regional vegetation restoration and ecological reconstruction.

To increase the efficiency of water acquisition and utilization, plants have developed a range of morphological and physiological adaptations over long-term evolution (Zhou et al., 2022; Ji et al., 2021). Among these, niche differentiation—especially differentiation in ecohydrological niches—is regarded as a key mechanism potentially promoting species coexistence and maintaining community structure and function (Samraoui et al., 2025). According to niche theory, competition among species that rely on similar resource-use patterns can hinder their long-term stable coexistence (Liu et al., 2024). In water-limited ecosystems, this differentiation is most prominently reflected in species-specific water-use strategies (Cory et al., 2022). In desert plant communities, two main modes of water use are commonly observed: (1) niche complementarity, in which different species, via root stratification or phenological differences, exploit water sources separated in space or time, thereby reducing direct competition for water (Ward et al., 2013); and (2) niche overlap, in which species depend on highly similar water sources, intensifying interspecific competition for limited water (Wu et al., 2024). Clarifying which mode dominates in a given community is crucial for understanding the assembly mechanisms of desert vegetation and predicting community responses to future environmental change.

Stable isotopes are effective natural tracers for identifying ecohydrological water sources (Han et al., 2023). Because different water sources (precipitation, soil water, groundwater) typically possess distinct isotopic signatures, stable isotope techniques have been widely applied in ecohydrological studies (Dong et al., 2024). A central advantage of this approach is that plant uptake and transport of water generally do not involve significant isotopic fractionation, and sampling can often be conducted in a minimally destructive manner. Among them, hydrogen (*δ*²H) and oxygen (*δ*¹^8^O) isotopes are key tracers for identifying plant water sources. By comparing the *δ*²H and *δ*¹^8^O values of plant xylem water with those of potential sources, it is possible to determine both the origin and the proportional contributions of water taken up by plants (Kühnhammer et al., 2023). Moreover, niche overlap indices derived from isotopic data can effectively quantify the similarity or divergence in water-resource use among species, thereby providing useful guidance for regional vegetation establishment and restoration (Weydmann-Zwolicka et al., 2022).

In the desert regions of northwestern China, *Nitraria tangutorum* and *Zygophyllum xanthoxylum* are two widely distributed and typical dominant shrubs. Both exhibit strong tolerance to salinity and drought and play irreplaceable ecological roles in reducing wind erosion, and stabilizing sandy surfaces (Cheng et al., 2024). Morphologically, *N. tangutorum* typically forms shrub clumps with curved or prostrate branches, well-developed root suckers, and relatively extensive horizontal spread, enabling it to rapidly occupy space and respond sensitively to rainfall events (Li et al., 2022). In contrast, *Z. xanthoxylum* usually forms spherical or hemispherical shrub clumps with broad canopies and a deep root system extending over a wide vertical range, allowing it to access deep soil water or groundwater (Fu et al., 2023; Xing et al., 2024); it is considered a classic deep-rooted xerophytic shrub. These traits highlight pronounced morphological differences between the two species.

Existing studies on the water-use characteristics of *N. tangutorum* and *Z. xanthoxylum* have mostly focused on single species, on contrasting habitats, or under specific stress conditions. Zhou et al. reported that *N. tangutorum* can use more surface soil water in spring, but shifts to deeper soil water or groundwater in summer and autumn (Zhou et al., 2013). Qin et al., working in the Badain Jaran Desert, found that *Z. xanthoxylum* primarily takes up water from soil deeper than 90 cm during the growing season (Qin et al., 2022a). However, it remains unclear how *N. tangutorum* and *Z. xanthoxylum* differ in their water-source partitioning patterns under the same habitat conditions. It is also unknown whether their water-use patterns are dynamically adjusted with seasonal changes, and whether the two shrubs exhibit niche complementarity or overlap in water use. These questions still lack systematic, *in situ* field evidence. Furthermore, relatively few studies on water use by desert shrub communities have continuously tracked the seasonal dynamics of plant water sources over an entire growing season, or combined Bayesian mixing models such as MixSIAR(Mixing Models for Stable Isotope Analysis in R) with ecohydrological niches metrics to quantitatively assess the degree of water-source sharing and segregation among species.

To address these gaps, this study focused on *N. tangutorum* and *Z. xanthoxylum* in the Tengger Desert. Through multiple *in situ* sampling campaigns during the growing season, we obtained hydrogen and oxygen stable isotope compositions of precipitation, soil water at different depths, groundwater, and xylem water of the two shrubs, and combined these with continuous soil moisture monitoring. On this basis, we constructed a multi-endmember tracing framework linking “precipitation–soil water–groundwater–plant water.” Using the MixSIAR Bayesian mixing model, we quantitatively resolved the contributions of different soil-depth water reservoirs and groundwater to the water use of the two shrubs across months, and characterized their ecohydrological niches with niche breadth and similarity indices.

Notably, because our field investigation was conducted in species-specific stands rather than fully mixed-species plots, the niche metrics are used here primarily to compare water-source use patterns under shared regional conditions, and any implications for interspecific interactions are discussed cautiously.

The specific objectives were to:

1. characterize the spatiotemporal distribution and isotopic signatures of different water reservoirs during the growing season and clarify the layered structure of soil water;
2. determine the seasonal composition of water sources for *N. tangutorum* and *Z. xanthoxylum* and compare differences and seasonal adjustments in their water-use strategies;
3. evaluate the degree of similarity and differentiation in water-source use between the two shrubs based on isotope-derived niche metrics, and discuss potential implications for water-use niche partitioning under shared regional conditions.

The results of this study deepen our understanding of adaptive strategies in desert plants, provide a scientific basis for species selection and configuration in ecosystem restoration, and have important practical implications for water resource management and ecological conservation in arid regions under ongoing global climate change.

## 2 Materials and methods

### 2.1 Study area

**Figure 1:**
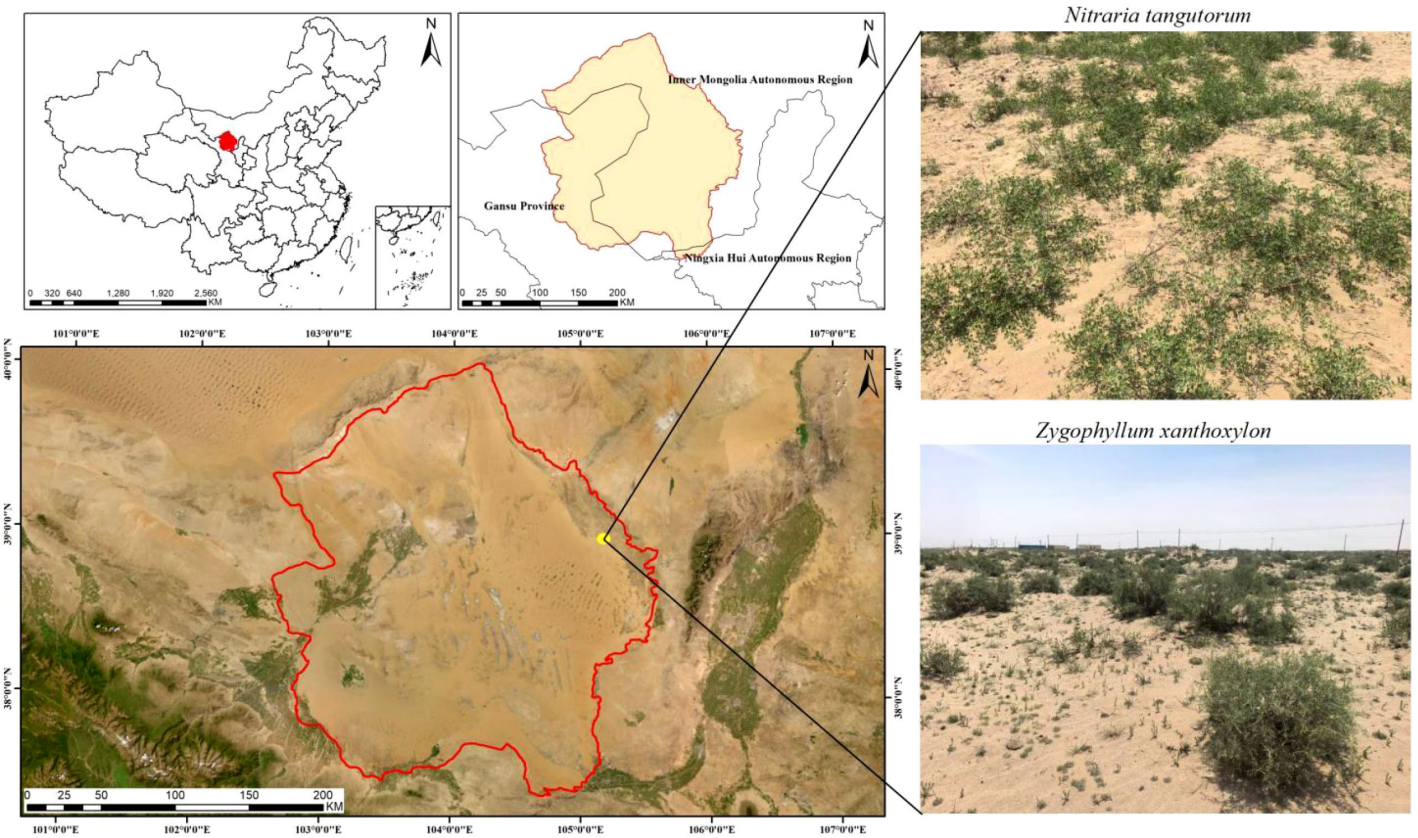
Geographic location of the study area.

**Figure 2:**
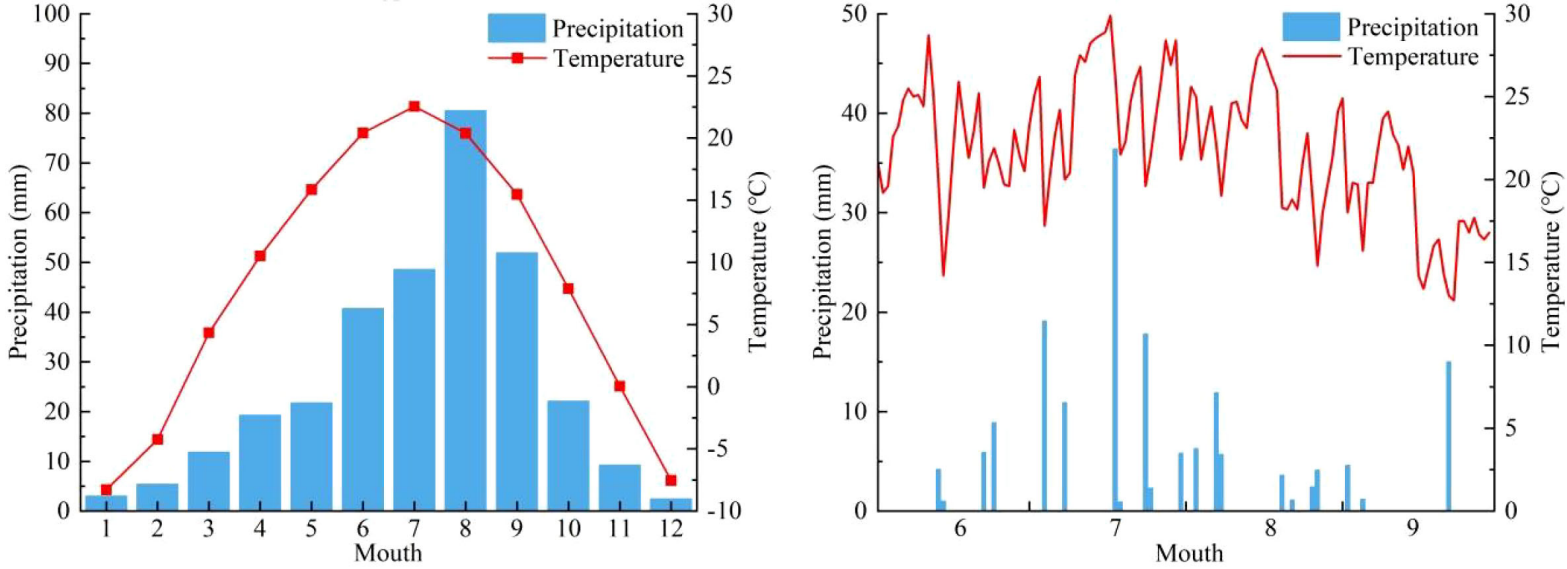
Variations in precipitation and air temperature in the study area. Caption: The left-hand figure shows changes in precipitation and temperature within the study area from 2015 to 2024; the right-hand figure shows changes in precipitation and temperature within the study area from June to September 2025.

The study area is located in the eastern part of the Tengger Desert in Northwest China, within the desert–oasis ecotone, at an elevation of 1200–1300 m (**Figure 1**). The region is characterized by a typical temperate continental climate. Long-term observational data indicate that annual precipitation is very limited, ranging from 71.44 to 116.60 mm, and is mainly concentrated between July and September (**Figure 2**). Evaporation far exceeds rainfall, with a mean annual evaporation of 2900–3300 mm. The area receives abundant solar radiation and strong evaporative demand, with a mean annual temperature of approximately 7.5–8.5 °C and >2800 hours of sunshine per year. Owing to the combined effects of scarce precipitation and intense evaporation, surface water resources in the study area are extremely limited. Measurements of groundwater depth indicate that the groundwater table at the study sites remained relatively stable at 10–30 m below the surface during the growing season. Soils in both shrub plots are dominated by aeolian sandy substrates, characterized by loose structure, low water-holding capacity, and low organic matter content. However, soil particle-size distributions differ between the two shrub communities: as shown in **Table 1**, soils in the *Z. xanthoxylum* plots generally have coarser particles than those in the *N. tangutorum* plots. These texture differences are important controls on infiltration and soil water retention and were therefore considered when interpreting inter-plot differences in soil moisture dynamics and isotope patterns. The vegetation is mainly composed of xerophytic and hyper-xerophytic shrubs, among which *N. tangutorum* and *Z. xanthoxylum* are the dominant and community-forming species in the region.

**Table 1:** Soil particle-size distribution in the *Nitraria tangutorum* and *Zygophyllum xanthoxylum* plots.

Plant	Clay (%) 0-2μm	Silt (%) 2-50μm	Sand (%)			
			Very fine sand (%) 50-100μm	Fine sand (%) 100-250μm	Medium (%) 250-500μm	Coarse sand (%) 500-1000μm
*Nitraria tangutorum*	0.04 ± 0.02	0.42 ± 0.07	6.09 ± 0.15	77.47 ± 0.27	15.96 ± 0.36	0.02 ± 0.26
*Zygophyllum xanthoxylum*	0.03 ± 0.01	0.29 ± 0.08	0.95 ± 0.02	29.87 ± 0.72	46.48 ± 0.24	22.27 ± 0.87

### 2.2 Sample collection

During the 2025 growing season (June–September), *Nitraria tangutorum* and *Zygophyllum xanthoxylum* were selected as the target species in the study area. For each shrub community type, three standard individuals with similar size and morphology were selected as target plants. The sample size (n = 3) was selected primarily to facilitate comparison of species-level differences in water-source use under similar regional environmental conditions, rather than to comprehensively characterize variability at the community scale. To minimize the influence of individual variation, the sampled plants within each shrub community were selected to be as similar as possible in size and morphology. Both shrub communities represent natural vegetation and are located in adjacent positions within the same study area, thereby minimizing differences in regional climatic conditions and background soil–hydrological settings. Specifically, the *N. tangutorum* plots are distributed in the northern part of the study area, where shrubs form small sand mounds with heights generally less than 30 cm, whereas the *Z. xanthoxylon* plots are located in the southern part of the area. A narrow transitional zone with mixed occurrence of the two shrubs exists between the two communities; however, mixed stands account for only a very small proportion of the local vegetation. Sampling sites were positioned in the central portions of each shrub community and were separated by a distance greater than 500 m, thereby minimizing potential interference from mixed vegetation and ensuring that sampled individuals represented species-specific stands rather than mixed plots. Nevertheless, we acknowledge that the limited number of sampled individuals may not fully capture the full range of intraspecific variability at the community scale; therefore, the results should be interpreted as comparative evidence of species-specific water-use patterns under shared site conditions.

A simple precipitation collection system consisting of a funnel and a rain gauge was installed in the study area. To reduce evaporative loss, ping-pong balls were placed on the funnel surface as a cover. After each rainfall event, precipitation samples were collected immediately, transferred into 15 mL glass vials, sealed with Parafilm, and kept cool with ice packs. The samples were promptly transported to the laboratory for hydrogen and oxygen stable isotope (*δ*²H, *δ*¹^8^O) analysis, and the date and amount of each rainfall event were recorded in detail.

For plant sampling, xylem samples were collected once per month from the three standard individuals in each plot. Several fully lignified, non-green twig segments (3–4 cm in length) were cut from each plant. The outer phloem tissue was removed, and the xylem segments were placed into 15 mL glass vials with sealed caps, wrapped with Parafilm, labeled, and then transported to the laboratory for subsequent water extraction and immediately stored at −20°C upon arrival.

Within a 1 m radius around each standard individual, three sampling points were selected for soil sampling. A hand-operated soil auger was used to collect soil at 20 cm intervals down to a depth of 200 cm, yielding 10 depth intervals in total. Soil from each depth was divided into two subsamples: one portion was placed into pre-weighed aluminum containers for determination of gravimetric soil water content; the other portion was stored in 50 mL sampling bottles, sealed with Parafilm, and immediately frozen at -20°C for subsequent water extraction and isotopic analysis.

In the vicinity of the experimental plots, the nearest monitoring well was selected as the groundwater sampling point. Before sampling, standing water in the well was pumped out, and then three parallel groundwater samples were collected. Groundwater was stored in 15 mL glass vials, sealed with Parafilm, and kept at 4 °C (in a portable cooler) until analysis and refrigerated at 4°C in the laboratory prior to isotope measurement.

Plant, soil, and groundwater samples were collected synchronously once per month from June to September. Each sampling campaign was carried out after at least three consecutive sunny days to ensure that a relatively stable state of plant water use was captured. In total, four sampling campaigns were conducted over the growing season, yielding a sufficient time series to characterize the seasonal dynamics of water use.

### 2.3 Sample processing and measurements

Soil water content was determined using the oven-drying method: soil samples in aluminum tins were dried at 105 °C to constant weight, and gravimetric water content was calculated from the difference between pre- and post-drying mass.

Plant xylem and soil samples stored in glass vials were subjected to water extraction using a cryogenic vacuum condensation system (Model LI-2000, LICA United Technology, China), with an extraction duration of 3 h. The *δ*²H and *δ*¹^8^O values of liquid water extracted from soil and plant samples, as well as precipitation samples, were then determined using a liquid water isotope analyzer (DLT-100, LGR, USA). Each sample was measured in triplicate and the mean value was used for subsequent analyses.

Isotopic compositions are expressed in per mil (‰) relative to Vienna Standard Mean Ocean Water (V-SMOW), and *δ*²H and *δ*¹^8^O are calculated as (Fry, 2015) (Equation 1):

(1) $$\delta \left(‰\right)=\frac{{\text{R}}_{\text{sample}}-{\text{R}}_{\text{standard}}}{{\text{R}}_{\text{standard}}}\times 1000$$

where Rsample and Rstandard represent the abundance ratios of the heavy to light isotopes (²H/¹H and ¹^8^O/¹^6^O) in the sample and the standard, respectively.

### 2.4 Isotopic-based source partitioning

To quantify the proportional use of different potential water sources by *N. tangutorum* and *Z. xanthoxylum*, we analyzed hydrogen and oxygen stable isotope data using the MixSIAR(Mixing Models for Stable Isotope Analysis in R) Bayesian mixing model(v3.1.12) implemented in R(v4.3.2). MixSIAR accounts for uncertainty in source and mixture isotope values and provides probabilistic (posterior) estimates of source contributions.

The model inputs included: (1) source data, i.e., *δ*²H and *δ*¹^8^O values of all potential water sources (soil water at different depths, groundwater, and precipitation); and (2) mixture data, i.e., *δ*²H and *δ*¹^8^O values of stem xylem water in *N. tangutorum* and *Z. xanthoxylum*. In this study, we assumed that plant water uptake does not induce significant isotopic fractionation, consistent with the common assumption in isotope-based plant water-source studies.

Model runs consisted of three Markov chain Monte Carlo (MCMC) chains, each with a length of 300,000 iterations and a burn-in of 200,000 iterations to ensure adequate convergence. Model convergence was assessed using the Gelman–Rubin diagnostic, with values < 1.05 indicating satisfactory convergence. The results are presented as the median and 95% credible intervals (CrI) of the posterior distributions of the proportional contributions from each water source.

### 2.5 Calculation of ecohydrological niche indices

Ecohydrological niche indices can be used to quantify similarity and differentiation in water-source use among species. In this study, we used Levins’ index to characterize ecohydrological niche breadth and the Proportional Similarity Index (PS index) to assess niche overlap. These indices provide a quantitative description of how broadly each species uses available water sources and how similar their source-use proportions are. The calculation formulas are as follows (Zhou H. J., et al., 2025; Stefan et al., 2016) (Equations 1, 2):

(2) $$\begin{array}{c} {\text{B}}_{\text{i}}=\frac{1}{\sum_{\text{j}=1}^{\text{r}}{\text{P}}_{\text{ij}}^{2}} \end{array}$$

where B_i_ denotes the ecohydrological niche breadth of species i, ranging from 0 to 1, with larger values indicating a broader niche; P_ij_ is the proportion of water used by species i from soil layer j; and r is the total number of soil layers (Equation 3).

(3) $$\begin{array}{c} \text{PS}=1-0.5\sum_{\text{j}=1}^{\text{r}}|\;{\text{P}}_{\text{ij}}-{\text{P}}_{\text{kj}}| \end{array}$$

where PS is the Proportional Similarity Index; larger PS values indicate that the two species share a higher proportion of the same water sources. P_ij_ and P_kj_ represent the proportion of water used by species i and k, respectively, from water source j, and r is the total number of water sources. Notably, because our field investigation was conducted in species-specific stands rather than mixed-species plots, these indices are interpreted primarily as comparative metrics of water-source use patterns under shared regional conditions, rather than as direct evidence of interspecific competition.

### 2.6 Data processing and statistical analysis

**Figure 3:**
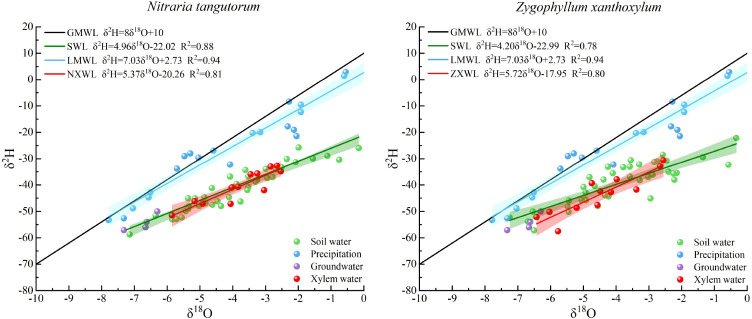
*δ*²H and *δ*¹^8^O values of different water sources during the study period and their fitted *δ*^2^H–*δ*¹^8^O relationships. Caption: GMWL, Global meteoric water line; SWL, Soil water line; LMWL, Local meteoric water line; NXWL, *Nitraria tangutorum* xylem water line; ZXWL, *Zygophyllum xanthoxylum* xylem water line. Differences in slopes and intercepts among fitted lines were tested using ANCOVA. SWL slopes and intercepts were significantly lower than the LMWL in both shrub plots (p < 0.001), whereas SWL slopes did not differ significantly between shrub plots (p = 0.149).

All raw data were first organized and preliminarily processed in Microsoft Excel 2019. Figures and tables were prepared using Origin 2022. The proportional contributions of different water sources to plant water use were calculated with a Bayesian mixing model (MixSIAR v3.1.12) implemented in the R environment (v4.3.2). Soil water content (SWC) differences between shrub plots across months and depths were evaluated using linear mixed-effects models. For *δ*^2^H–*δ*^18^O relationships (**Figure 3**), differences in slopes and intercepts among fitted lines (LMWL, SWL, and XWL) were tested using ANCOVA, reporting parameter estimates with standard errors and p-values.

## 3 Results

### 3.1 Characteristics of precipitation, air temperature, and soil moisture dynamics

This study focused on the period from June to September 2025 as the core study window, during which approximately 70% of the annual precipitation occurs in the region (based on the 2015–2024 climatic record) (**Figure 2**). This indicates that the observation period captures the main hydroclimatic conditions of the local growing season. Observations for 2025 show that hydrothermal conditions were characterized by concurrent peaks in precipitation and air temperature during this period. Precipitation in July was the highest (93.2 mm), with the largest single rainfall event (36.4 mm) recorded on 18 July; August ranked second (35.1 mm), while precipitation in June and September was relatively low (20.0 mm and 20.8 mm, respectively). Mean monthly air temperature increased from June onward, peaked in July (24.7 °C), and then declined to 18.3 °C in September. This seasonal alignment of precipitation and temperature indicates that July–August represent the main hydroclimatic window for soil moisture recharge during the growing season. Nevertheless, because this study covered only one growing season, the observed patterns should be interpreted as reflecting plant water-use responses under the hydroclimatic conditions of 2025 rather than stable long-term ecological strategies, and they may not fully capture interannual variability.

**Figure 4:**
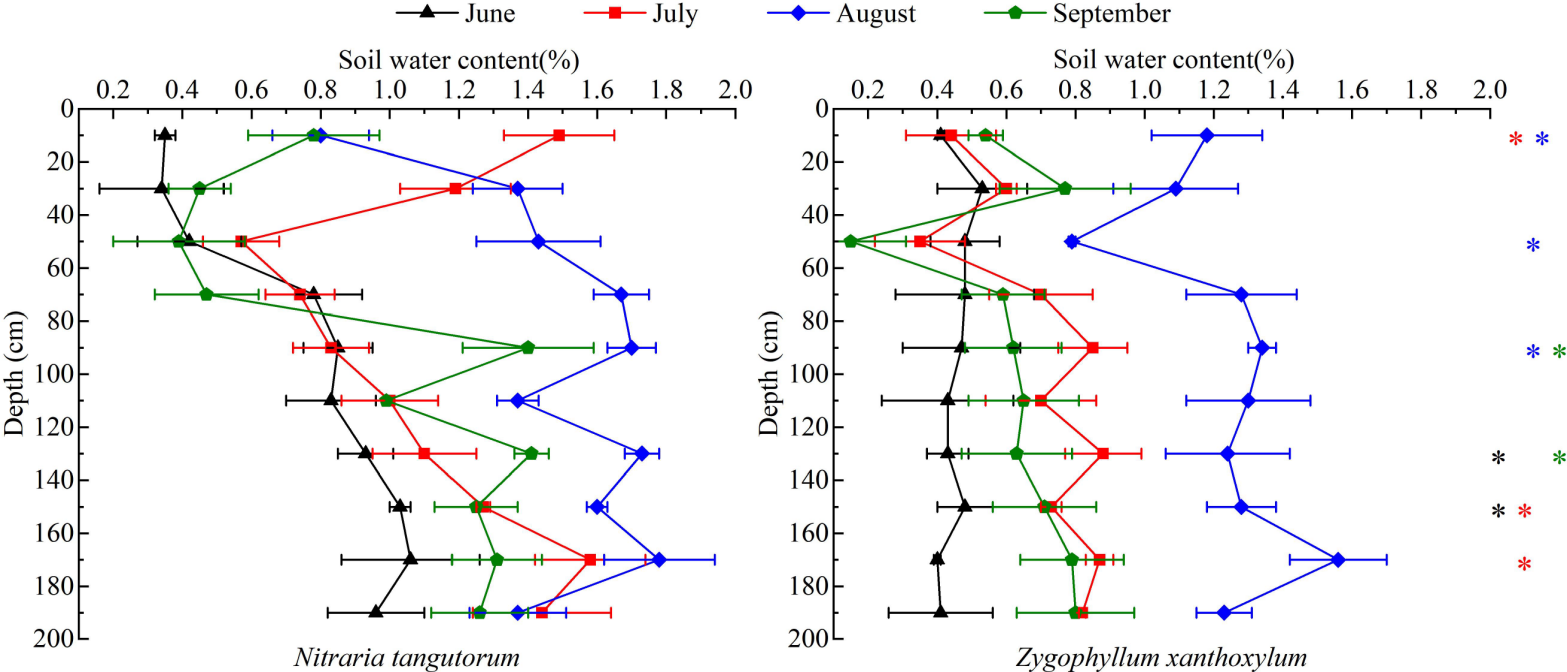
Spatiotemporal dynamics of soil water content (SWC)(0–200 cm) in *Nitraria tangutorum* and *Zygophyllum xanthoxylum* stands. Caption: Points represent mean ± SE (n = 3). Colored asterisks indicate significant interspecific differences between the two shrub species at the same sampling month and soil depth (p < 0.05).

As shown in **Figure 4**, soil water content (SWC) beneath both shrub communities exhibited clear vertical stratification and clear seasonal dynamics during June–September. Along the soil profile, SWC in the *N. tangutorum* plots was generally higher in the deep layer (120–200 cm) than in the shallow layer (0–40 cm), whereas the vertical gradient in the *Z. xanthoxylum* plots was comparatively weak, with smaller moisture differences among depth intervals. Seasonally, SWC in the *N. tangutorum* plots reached its growing-season maximum in July or August, and the deep-layer maximum most frequently occurred in August. In contrast, SWC in the *Z. xanthoxylum* plots peaked predominantly in August, showing a consistent pattern across soil layers. Overall, SWC was generally low in June, increased markedly in July–August in response to rainfall inputs, and declined in most layers by September. Across the majority of sampling dates and depths, SWC in the *N. tangutorum* plots was higher than that in the *Z. xanthoxylum* plots, particularly in the middle-to-deep layers (40–200 cm) in August (p < 0.05). These patterns suggest contrasting soil-water environments between the two shrubs communities, potentially reflecting combined effects of shrub canopy structure, litter accumulation, and soil texture differences (**Table 1**). Specifically, the relatively higher SWC under *N. tangutorum* may indicate greater near-surface moisture retention, whereas the weaker vertical gradient under *Z. xanthoxylum* is consistent with more uniform moisture depletion across depths. These interpretations should be considered indicative, because root distribution and *in situ* evaporation were not directly quantified.

### 3.2 Isotopic characteristics of different water sources

The slopes and intercepts of the fitted lines for *δ*²H and *δ*¹^8^O differed among the various water sources (**Figure 3**), and differences in regression parameters were tested using ANCOVA. Based on the hydrogen and oxygen isotope data of precipitation collected during the experiment, the local meteoric water line (LMWL) in the study area was established as: *δ*²H = 7.03*δ*¹^8^O + 2.73(R² = 0.94)(n=22), The estimated slope and intercept were 7.03 ± 0.40 and 2.73 ± 1.82(± SE), respectively. The LMWL slope was lower than that of the global meteoric water line (GMWL: *δ*²H = 8*δ*¹^8^O + 10), which is consistent with evaporative effects on precipitation under arid atmospheric conditions.

The soil water lines (SWL) for the *N. tangutorum* and *Z. xanthoxylum* plots were:*δ*²H = 4.96*δ*¹^8^O – 22.02(R² = 0.88)(n=40), slope ± SE = 4.96 ± 0.29 and intercept ± SE = −22.01 ± 1.21 and *δ*²H = 4.20*δ*¹^8^O – 22.99(R² = 0.72)(n=40), respectively. The estimated slopes and intercepts were 4.96 ± 0.29 and −22.01 ± 1.21 for the *N. tangutorum* plot, and 4.20 ± 0.43 and −22.99 ± 1.79 for the *Z. xanthoxylum* plot (± SE).For both shrub communities, the SWL slopes and intercepts were significantly lower than those of the LMWL (ANCOVA slope tests: *N. tangutorum* SWL vs. LMWL, p = 5.56 × 10^-5^; *Z. xanthoxylum* SWL vs. LMWL, p = 1.04 × 10^-5^; intercept differences: p < 0.001 for both comparisons), indicating that soil water experienced stronger evaporative enrichment than precipitation. The SWL for the *N. tangutorum* plot showed a higher slope and better goodness of fit than that for the *Z. xanthoxylum* plot, although the SWL slope difference between shrub plots was not statistically significant (p = 0.149), suggesting a tendency toward relatively weaker evaporation of soil water, whereas the lower slope of the SWL in the *Z. xanthoxylum* plot was consistent with pointed to more intense soil water evaporation.

The xylem water lines for *N. tangutorum* and *Z. xanthoxylum* were *δ*^2^H = 5.37*δ*^18^O−20.26 (R^2^ = 0.81)(n = 12) and *δ*^2^H =5.72*δ*¹^8^O−17.95(R^2^ = 0.80)(n = 12), respectively. In both species, xylem isotopic compositions were more closely aligned with soil water and were clearly offset from the isotopic signatures of precipitation and groundwater, indicating that *N. tangutorum* and *Z. xanthoxylum* relied primarily on soil water during the growing season. At the same time, the positions of xylem water isotopes for the two species along the soil water line differed, implying that *N. tangutorum* and *Z. xanthoxylum* may access soil water from different depths, thereby achieving ecohydrological niche differentiation in water use under the studied conditions.

### 3.3 Plant water-use patterns

Soil water at different depths serves as the direct water source for plants, whereas precipitation is an indirect source. In general, root water uptake of plants in arid regions does not cause appreciable fractionation of *δ*¹^8^O. Accordingly, the oxygen isotopic composition (*δ*¹^8^O) of stem xylem water is commonly assumed to reflect that of the water sources taken up by roots. On this basis, comparison of stem water *δ*¹^8^O with the *δ*¹^8^O values of soil water at different depths can provide a qualitative indication of the likely depths of plant water uptake. In this study, the term “intersection” refers to the depth at which the *δ*¹^8^O value of stem xylem water most closely matches that of soil water in the vertical profile, suggesting that this layer may have contributed importantly to plant water uptake. However, this approach provides only qualitative evidence, whereas the proportional contributions of different water sources were further quantified using the MixSIAR model.

**Figure 5:**
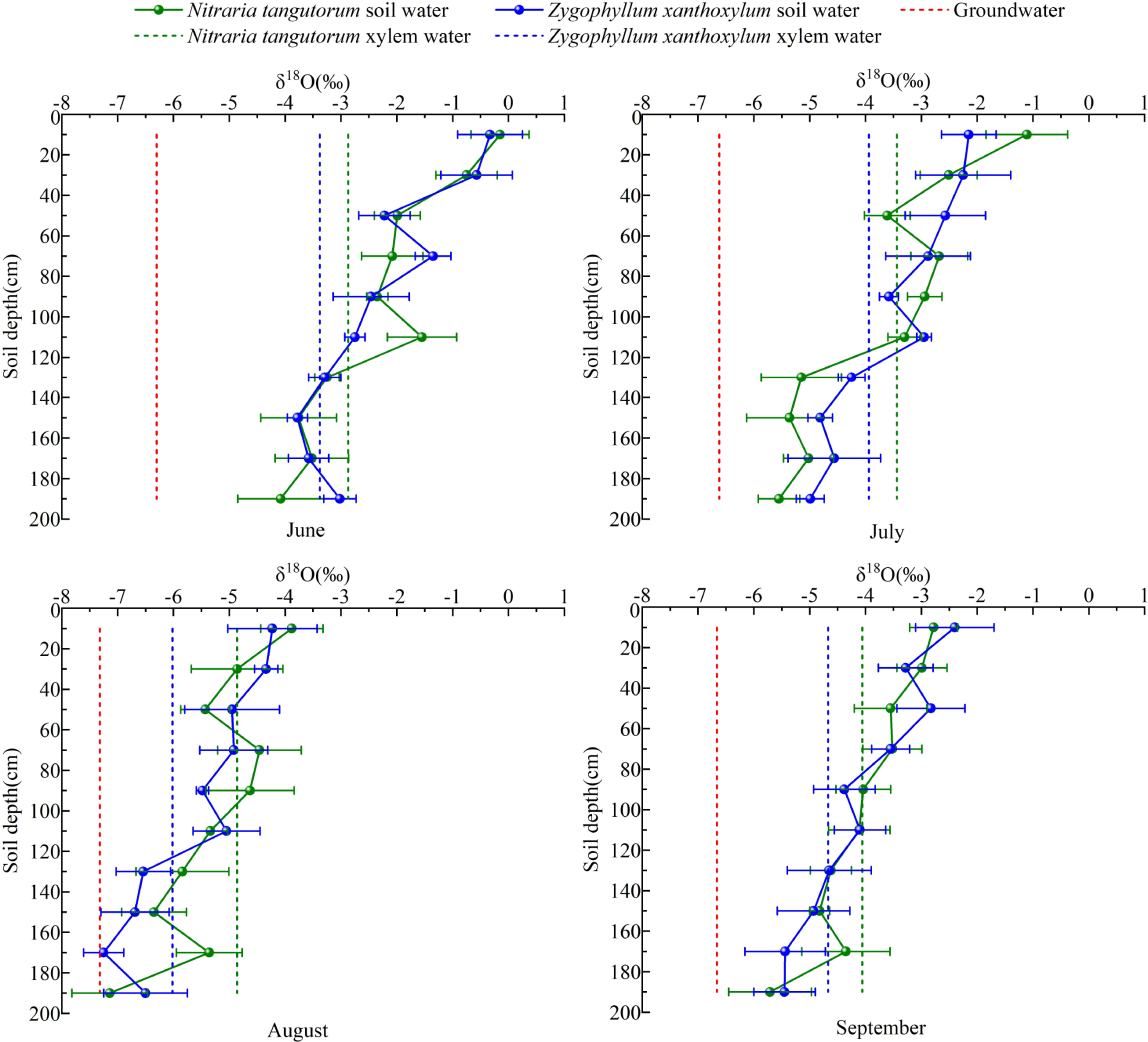
Hydrogen and oxygen isotopic characteristics of plant stem water, soil water, and groundwater.

Based on the vertical *δ*¹^8^O profile of the soil, we analyzed the water-use strategies of *N. tangutorum* and *Z. xanthoxylum* at 20 cm soil-depth intervals. The results showed (**Figure 5**) that in June, the *δ*¹^8^O values of stem water in both species intersected with those of deep soil water. In July, the *δ*¹^8^O signal of *N. tangutorum* stem water intersected with soil water in the 40–60 cm and 100–120 cm layers, whereas that of *Z. xanthoxylum* intersected with soil water in the 120–140 cm layer. In August, *N. tangutorum* stem water intersected with soil water from the 20–40, 60–80, and 80–100 cm layers, whereas *Z. xanthoxylum* stem water intersected with soil water from the 120–140 cm layer. In September, the *δ*¹^8^O of *N. tangutorum* stem water aligned with soil water in the 80–100 cm layer, whereas *Z. xanthoxylum* again matched soil water in the 120–140 cm layer.

The fact that the intersection depths between stem water and soil water *δ*¹^8^O shifted among months indicates that the water uptake depths of *N. tangutorum* and *Z. xanthoxylum* vary seasonally under the observed field conditions.

**Figure 6:**
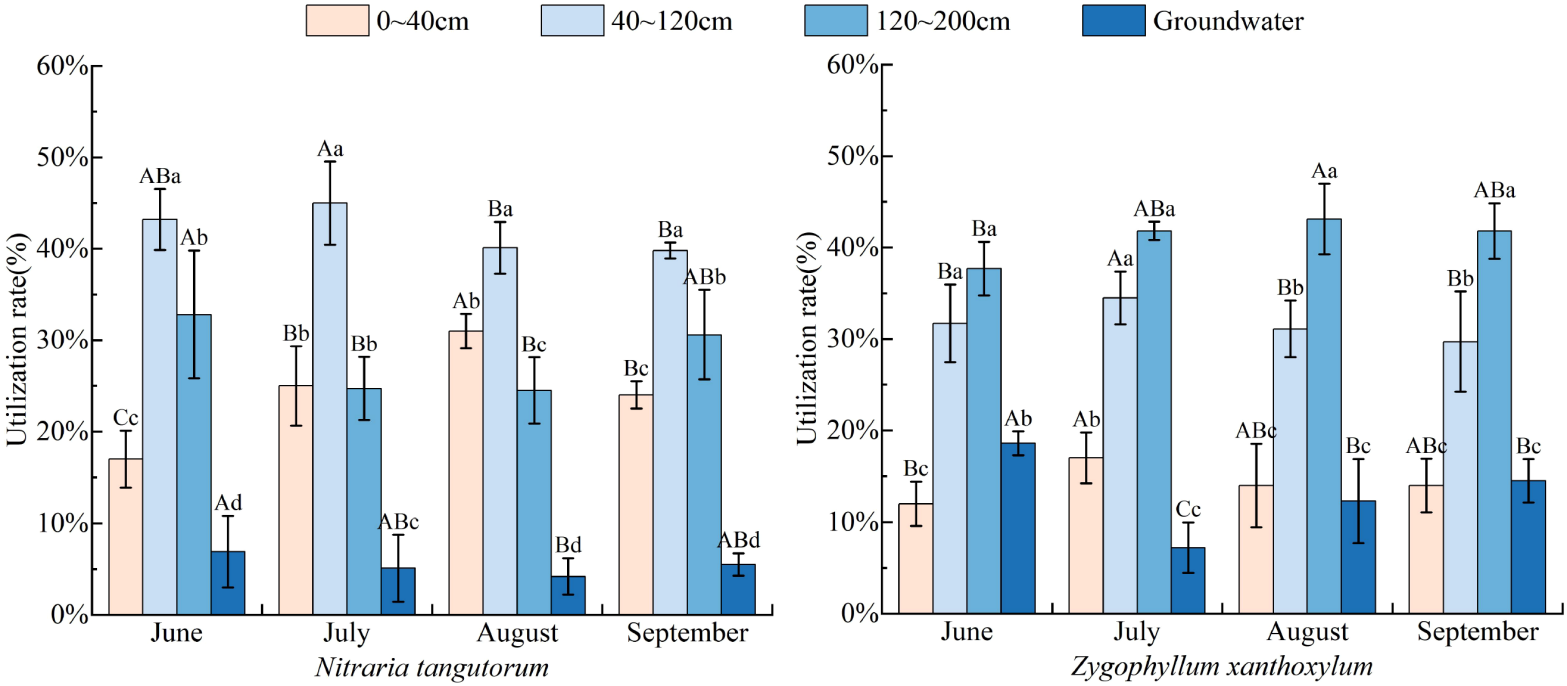
Proportional contributions of different water sources to plant water use based on the MixSIAR model.

Results of water-source partitioning based on the MixSIAR model (**Figure 6**) show that *N. tangutorum* and *Z. xanthoxylum* exhibit contrasting interspecific differences and seasonal adjustments in their water-use strategies. Overall, *N. tangutorum* relied predominantly on middle (40–120 cm) and deep (120–200 cm) soil water in the early growing season (June), with these sources together contributing 76.0%. As the season progressed, it gradually increased its use of shallow (0–40 cm) soil water, reaching a maximum contribution of 31.1% in August, and then shifted back in September to mainly using middle (39.8%) and deep (30.6%) soil water. This pattern indicates a pronounced seasonal adjustment in the relative contributions of water sources used by *N. tangutorum*.

In contrast, *Z. xanthoxylum* followed a different utilization pattern: it already depended on deep soil water in June (37.7%), enhanced its use of soil water across all depths in July, with deep soil water contributing up to 41.6%, and maintained a high proportion of deep water use in August and September (42.9% and 41.8%, respectively). This suggests that *Z. xanthoxylum* tends to adopt a stable, deep-soil–water-based strategy.

For both species, the contribution of groundwater was relatively low (4.2%–6.9% for *N. tangutorum* and 7.2%–18.6% for *Z. xanthoxylum*), indicating that their water supply was dominated by soil water rather than groundwater. Notably, during the main rainy period (July–August), both species increased their use of shallow soil water, suggesting sensitivity to seasonal precipitation inputs and the ability to exploit newly replenished, shallow soil water at the monthly scale.

### 3.4 Dynamics of ecohydrological niches

In arid and semi-arid ecosystems, plant water use commonly exhibits strong temporal and vertical heterogeneity. As shown in **Table 2**, the niche breadth of *N. tangutorum* and *Z. xanthoxylum* followed a pattern of first decreasing and then increasing over the growing season, with ENB values remaining >3 in all months. The two species exhibited the most similar niche breadths in July and August, whereas the difference between them was greatest in June.

**Table 2:** Ecohydrological niche breadth (ENB) and Proportional Similarity (PS) index of water use for the two shrub species.

Index	Category	Month			
		6	7	8	9
ENB	*N. tangutorum*	3.05	3.03	3.13	3.19
	*Zygophyllum xanthoxylum*	3.43	3.07	3.17	3.29
PS	0-40cm Soil water	0.97	0.96	0.91	0.95
	40-120cm Soil water	0.94	0.95	0.96	0.95
	120-200cm Soil water	0.98	0.91	0.91	0.94
	Groundwater	0.94	0.99	0.96	0.96
	Soil water and Groundwater	0.83	0.81	0.73	0.80

In terms of the Proportional Similarity (PS) of different water sources, the two species showed the highest similarity in their use of deep soil water (120–200 cm) in June (PS = 0.98). In July and September, the highest similarity occurred in groundwater use (PS = 0.99 and 0.96, respectively), while in August the greatest similarity was observed for the use of middle-layer soil water and groundwater (both PS = 0.96). Overall, the PS index for soil water and groundwater use between the two species was lowest in August (0.73) and highest in June (0.83), indicating a seasonal shift in the degree of similarity in their water-source use patterns. Given that the two shrub communities were sampled in species-specific stands rather than mixed-species plots, PS is interpreted here as a comparative metric of water-source use similarity under shared regional conditions, rather than as direct evidence of interspecific competition.

## 4 Discussion

### 4.1 Patterns of soil moisture and stable isotope composition

By examining the spatiotemporal patterns of soil moisture and the hydrogen and oxygen stable isotope signatures of soil water under typical shrub stands in the Tengger Desert, this study shows that the soil environment in the study area is extremely dry, with low water content and pronounced spatial–temporal gradients. At the same time, soil water isotopes are systematically enriched relative to the local meteoric water line, revealing strong evaporative redistribution processes and providing a necessary basis for subsequent water-source partitioning.

In terms of soil moisture patterns, shallow soil water exhibited a seasonal trajectory of “low–high–slightly decreasing” over the growing season, being lowest in June and showing a modest recovery in August during the concentrated rainfall period. In contrast, deep soil water was much more stable over time. This suggests that precipitation infiltrates rapidly through the sandy substrate but, constrained by high evaporation and low water-holding capacity, only a fraction of the infiltrated water is “stored” in the middle and deeper layers. Compared with the *Z. xanthoxylum* plots, soil water content in the 0–200 cm profile was generally higher in the *N. tangutorum* plots (**Figure 4**). This inter-plot contrast is consistent with microenvironmental differences associated with shrub canopy cover, litter accumulation, and soil texture (**Table 1**), which can influence near-surface evaporation and infiltration in drylands (Tetzlaff et al., 2021).

The isotopic data further elucidate the mechanisms of soil water cycling. The slope of the local meteoric water line (LMWL) is lower than that of the global meteoric water line, and the slopes of the soil water lines (SWL) are in turn lower than that of the LMWL. This pattern indicates evaporative fractionation during infiltration and residence in the upper soil layers, leading to enrichment of shallow soil water in *δ*²H and *δ*¹^8^O, whereas middle and deep soil water is closer to the LMWL. The SWL for the *N. tangutorum* plots showed a slightly higher slope and better goodness of fit than that for the *Z. xanthoxylum* plots, although the slope difference was not statistically significant, suggesting a tendency toward weaker evaporative modification of soil water in the former. This tendency is consistent with the higher SWC observed under *N. tangutorum* (**Figure 4**), although direct measurements of evaporation and root distribution were not available in this study.

The isotopic signatures of xylem water in both species plot close to the soil water line and are clearly offset from the precipitation and groundwater end-members, further supporting the conclusion that the two shrubs mainly depend on soil water rather than directly using precipitation or groundwater. This is consistent with a fundamental assumption in classic isotope-based studies of plant water sources (Qiu et al., 2023). More importantly, xylem water in *N. tangutorum* is closer to shallow–middle soil water, whereas that in *Z. xanthoxylum* aligns more with middle–deep soil water, indicating vertical partitioning of water-use niches along the soil profile. Here, the inference of potential uptake depth from the “intersection” or isotopic matching between xylem water and soil water is based on the widely used assumption that root water uptake causes little or no appreciable oxygen isotope fractionation. Therefore, similarity between xylem-water *δ*¹^8^O and soil-water *δ*¹^8^O at a given depth can be used as a qualitative indicator of likely water uptake from that layer. However, such isotopic matching provides only qualitative evidence and should not be interpreted as an exact one-to-one estimate of source contribution; the quantitative partitioning of water sources in this study was therefore further assessed using the MixSIAR model. These results provide a basis for interpreting interspecific differences in water-source use patterns and demonstrate that isotopic gradients can trace water transfer pathways along the soil–plant continuum even under extremely arid conditions (Tetzlaff et al., 2021).

Overall, the combined evidence from soil moisture and isotopes indicates the presence of a vertically structured soil water system in the study area, consisting of a strongly evaporative shallow zone, a transitional middle zone, and a relatively stable deep reservoir. Within this layered system, the two shrub species exhibited contrasting depth preferences in water uptake, which may contribute to hydrological niche differentiation under shared regional conditions. At the same time, because the analysis was based on one growing season and a limited number of sampled individuals per shrub stand, these depth-related patterns are best interpreted as comparative evidence of dominant species-level water-use tendencies under the observed site conditions, rather than as complete representations of all possible temporal and individual variability.

### 4.2 Differences in plant water sources and water-use relationships

By analyzing the seasonal dynamics of water-source composition and water-use strategies of *N. tangutorum* and *Z. xanthoxylum* during the growing season, and combining the results of the isotopic direct-comparison approach with those from the MixSIAR model, this study shows that both shrubs rely primarily on soil water. However, *N. tangutorum* exhibits more pronounced seasonal switching of water sources and an “opportunistic” capacity to exploit newly recharged shallow soil water, whereas *Z. xanthoxylum* depends persistently on middle–deep soil water and a small fraction of groundwater, reflecting a more conservative and stable water-use pattern.

From the perspective of the direct-comparison method, the isotopic composition of xylem water in *N. tangutorum* clustered around the middle–deep soil water end-members in June. As precipitation became concentrated in July–August, its isotopic signal shifted toward the shallow soil-water domain, before gradually returning to the middle soil layers in September. In contrast, xylem water of *Z. xanthoxylum* remained largely within the isotopic range between deep soil water and groundwater throughout the growing season, with only slight upward shifts to shallower layers during the peak growth and rainfall period in July–August. This pattern aligns well with the highly plastic water-use strategies reported for Nitraria species under varying soil textures and hydrological conditions (Qiu et al., 2023; Gu et al., 2025) whereas *Z. xanthoxylum* resembles other xerophytic trees and shrubs with deep-rooted, deep-water-dependent strategies (Zhou H. et al., 2025).

Quantitative source partitioning by MixSIAR further reinforces these findings: *N. tangutorum* mainly relied on water from the 40–200 cm profile in the early growing season, then, during the concentrated rainfall period, the contribution of shallow (0–40 cm) soil water increased markedly, before the plant shifted back to a pattern dominated by middle–deep sources. *Z. xanthoxylum*, by contrast, consistently used soil water from 120–200 cm in all months, with groundwater contributions being slightly higher than those of *N. tangutorum* but still clearly subordinate to soil water. MixSIAR provides probabilistic estimates of source contributions while accounting for uncertainty in source and mixture isotope values (Stock and Semmens, 2016). In this study, the key conclusions derived from the direct-comparison method and from MixSIAR—such as the “main water uptake depth” and the “peak period of shallow-water use”—are highly consistent, indicating strong robustness of the results. Nevertheless, because the direct-comparison approach is inherently qualitative and monthly sampling may not fully capture short-lived post-rainfall shifts, the inferred timing and magnitude of water-source switching should be interpreted with appropriate caution.

Despite the relatively shallow groundwater table, the direct contribution of groundwater to the water use of both shrubs was limited and only slightly higher in *Z. xanthoxylum*. This pattern is consistent with syntheses at the global scale, which show that groundwater use by vegetation is widespread but typically contributes only a modest fraction of total plant water uptake (Evaristo and McDonnell, 2017). It also provides a useful contrast to studies in lacustrine-basin settings where *N. tangutorum* has been shown to switch between deep soil water and groundwater in response to groundwater-table fluctuations (Qiu et al., 2023; Qin et al., 2023).

Temporally, both shrubs increased their use of shallow soil water during the main rainy period in July–August, indicating that even under extremely arid conditions, desert shrubs retain the capacity to respond to seasonal precipitation inputs at the monthly sampling scale. This is consistent with previous findings for Nitraria species, which are characterized as being highly sensitive to rainfall events and capable of flexibly exploiting small and transient water sources (Gu et al., 2025). The key difference lies in strategy: *N. tangutorum* substantially increases its reliance on shallow soil water to “capture” the transient water reservoir created by rainfall, while *Z. xanthoxylum* makes only moderate adjustments in uptake depth and maintains its dependence on deeper, more stable water. This contrast is consistent with functional differentiation in water-source use between the two shrubs under the studied conditions (Zhou H. et al., 2025). Because the observation period from June to September encompasses the main rainy season and approximately 70% of the annual precipitation in the study area, the seasonal shifts reported here are likely representative of the principal hydroclimatic window of plant growth and soil-water recharge. However, these results still reflect the specific hydroclimatic conditions of 2025 and should not be overgeneralized as fixed long-term ecological strategies without validation from multi-year observations.

### 4.3 Similarity and differentiation in water-source use

The results of ecohydrological niche breadth and the Proportional Similarity (PS) index show that both shrub species occupied relatively broad ecohydrological niches throughout the growing season, but that niche overlap weakened during the concentrated rainfall period. This pattern reflects seasonal shifts in the degree of similarity in water-source use along the soil profile.

In terms of niche breadth, the values for *N. tangutorum* and *Z. xanthoxylum* were greater than 3 in all months, indicating that both species drew on a wide spectrum of water sources, ranging from shallow to deep soil water and even groundwater. This highlights the “multi-endmember, broad-spectrum” water-use strategy typical of desert shrubs (Dong et al., 2022). However, the niche breadth of both species narrowed temporarily in July–August and then increased again in September, suggesting that during the peak rainfall period both species tended to adopt more “specialized” water-use strategies to enhance their access to key water reservoirs.

The PS index further clarifies the scale at which similarity and differentiation occur. Deep soil water and groundwater exhibited relatively high PS values in most months, indicating that the two shrubs shared and overlapped to some extent in these more stable water reservoirs. This is consistent with the “passive co-use” mechanism, whereby deep-rooted shrubs under prolonged drought are forced to jointly depend on deep water stores (Evaristo and McDonnell, 2017). Nevertheless, the overall PS index for combined soil water and groundwater was highest in June and lowest in August, implying that during the strongest precipitation pulses, *N. tangutorum* and *Z. xanthoxylum* achieved greater water-use niche differentiation by adjusting their reliance on shallow and middle soil layers: *N. tangutorum* increased its use of newly recharged shallow water, whereas *Z. xanthoxylum* maintained its dependence on deeper water, thereby partitioning the water-source space in both time and depth (Gu et al., 2025; Zhou H. et al., 2025).

Overall, the two shrubs exhibited contrasting depth preferences and seasonal adjustments in water-source use, which are consistent with ecohydrological niche differentiation along the soil profile (Dong et al., 2022). Notably, because the two shrub communities were sampled in species-specific stands rather than mixed-species plots, these niche metrics are interpreted primarily as comparative indicators of water-source use patterns under shared regional conditions, and not as direct evidence of interspecific competition or long-term stabilizing coexistence mechanisms. In addition, the sample size in each shrub community was limited (n = 3), and although individuals were selected to be as similar as possible in size and morphology, this design may not fully capture the full range of intraspecific variability at the community scale. Therefore, the niche metrics reported here should be interpreted cautiously as reflecting dominant species-level tendencies rather than complete community-level variability.

These insights offer a basis for species selection and configuration in desertification control and vegetation restoration: in areas with relatively shallow groundwater tables, combining deep-rooted species (e.g., *Z. xanthoxylum*) with shallow- to intermediate-rooted, rainfall-sensitive shrubs (e.g., *N. tangutorum*) can enhance the buffering capacity of the system against climatic variability while maintaining limited groundwater dependence under the studied conditions (Zhou H. et al., 2025).

It should be noted, however, that this study has several limitations that warrant further work. First, the observation period covered only a single growing season, making it difficult to capture interannual variation in water-use patterns under extremely dry or extremely wet conditions. Although the June–September study window encompasses the main rainy season and approximately 70% of the annual precipitation based on the 2015–2024 climatic record, plant water-use strategies in arid environments may still vary substantially among years because of differences in precipitation amount, timing, and infiltration depth. Therefore, the patterns observed here should be interpreted as responses under the hydroclimatic conditions of 2025 rather than as fixed long-term ecological strategies. Second, soil and plant water were extracted using traditional cryogenic vacuum distillation, and we assumed negligible isotopic fractionation during root water uptake. Recent studies have shown that different extraction methods and internal tissue water pools may introduce systematic biases (Tetzlaff et al., 2021; Gai et al., 2023), thereby increasing the uncertainty of quantitative source partitioning. Even so, the key features identified here—such as the “main water uptake depth” and the “direction of seasonal shifts”—were highly consistent across methods. Third, the monthly sampling design limits our ability to resolve rapid post-rainfall dynamics; event-based sampling would be needed to quantify pulse-response timing and duration.

Taken together, our results demonstrate that in the typical shrubland ecosystems of the Tengger Desert, a distinctly layered soil-water reservoir, combined with differentiated water-use strategies and seasonal hydrological niche separation, is associated with contrasting water-source use patterns of the two dominant shrubs during the growing season. This conclusion deepens our understanding of vegetation–water coupling mechanisms in deserts from an integrated hydrological and ecological perspective, and points toward future research directions involving multi-year observations, multi-isotope approaches, and explicit incorporation of root functional traits.

## 5 Conclusions

This study investigated the water-use strategies of *Nitraria tangutorum* and *Zygophyllum xanthoxylum* in the Tengger Desert. The main conclusions are as follows:

1. Soil moisture in the study area was generally extremely low and exhibited a vertically stratified pattern characterized by strong evaporation in the shallow layer, a transitional middle layer, and a relatively stable deep layer. Soil water isotopes were clearly enriched relative to the meteoric water line, and the differences in SWL slopes between the *N. tangutorum* and *Z. xanthoxylum* plots suggest a tendency that vegetation structure and microenvironment jointly shape the spatiotemporal distribution and evaporative behavior of soil water(noting that the SWL slope difference between shrub plots was not statistically significant).
2. Both *N. tangutorum* and *Z. xanthoxylum* relied primarily on soil water, with only limited direct use of groundwater. At the seasonal scale, *N. tangutorum* showed a “deep-to-shallow-then-back-to-deep” pattern of water-source switching and was more strongly associated with increased use of shallow soil water during the main rainy period (July–August) at the monthly sampling scale. In contrast, *Z. xanthoxylum* persistently depended on middle–deep soil water and a small amount of groundwater, reflecting a stable and conservative water-use strategy. These contrasting strategies indicate functional differentiation in water-source use between the two shrubs under the studied conditions.
3. Ecohydrological niche analysis showed that both shrubs maintained relatively broad water-use niches throughout the growing season. However, water-source overlap decreased during the concentrated rainfall period, accompanied by pronounced differentiation in the use of shallow and middle soil water. This seasonal shift indicates greater differentiation in water-source use along vertical and temporal dimensions during the main rainy period. Because the study was conducted in species-specific stands rather than mixed-species plots, these niche metrics are interpreted as comparative indicators of water-source use patterns under shared regional conditions, rather than as direct evidence of interspecific competition or coexistence mechanisms.

## Data Availability

The original contributions presented in the study are included in the article/supplementary material. Further inquiries can be directed to the corresponding author.
